# Effects of nasal inflammation on the olfactory bulb

**DOI:** 10.1186/s12974-022-02657-x

**Published:** 2022-12-09

**Authors:** Brandon J. LaFever, Fumiaki Imamura

**Affiliations:** Department of Pharmacology, Penn State College of Medicine, 500 University Dr., Hershey, PA 17033 USA

**Keywords:** Inflammation, Lipopolysaccharide, Olfaction, Olfactory bulb, Rhinosinusitis

## Abstract

Sinonasal diseases, such as rhinosinusitis, affect up to 12% of individuals each year which constitutes these diseases as some of the most common medical conditions in the world. Exposure to environmental pathogens and toxicants via the nasal cavity can result in a severe inflammatory state commonly observed in these conditions. It is well understood that the epithelial and neuronal cells lining the olfactory mucosa, including olfactory sensory neurons (OSNs), are significantly damaged in these diseases. Prolonged inflammation of the nasal cavity may also lead to hyposmia or anosmia. Although various environmental agents induce inflammation in different ways via distinct cellular and molecular interactions, nasal inflammation has similar consequences on the structure and homeostatic function of the olfactory bulb (OB) which is the first relay center for olfactory information in the brain. Atrophy of the OB occurs via thinning of the superficial OB layers including the olfactory nerve layer, glomerular layer, and superficial external plexiform layer. Intrabulbar circuits of the OB which include connectivity between OB projection neurons, OSNs, and interneurons become significantly dysregulated in which synaptic pruning and dendritic retraction take place. Furthermore, glial cells and other immune cells become hyperactivated and induce a state of inflammation in the OB which results in upregulated cytokine production. Moreover, many of these features of nasal inflammation are present in the case of SARS-CoV-2 infection. This review summarizes the impact of nasal inflammation on the morphological and physiological features of the rodent OB.

## Introduction

On a daily basis, we are exposed to a variety of chemicals and environmental agents in the air, some of which are seemingly harmless while others have the potential to cause serious diseases. Bacteria, viruses, fungal toxicants, and various allergens are some examples of volatile agents that can be inhaled and cause pathological changes to the body. When inhaled, these various agents can enter the body through the nasal cavity and induce inflammation of the paranasal sinuses and olfactory mucosa (OM) which consists of the olfactory epithelium (OE) and the underlying lamina propria [1, 2]. Inflammatory diseases of the nasal cavity are collectively referred to as “sinonasal diseases”. Sinonasal diseases including rhinitis, sinusitis, and rhinosinusitis are some of the most common medical conditions in the world [3]. The prevalence of these conditions increases annually [4]. Some sinonasal diseases, such as rhinosinusitis, affect between 8 and 9% of individuals in Asian countries [5, 6], 11% in European countries [7], and up to 12.5% of the United States’ population which is approximately 40 million individuals [8, 9].

Symptoms of sinonasal diseases can be rather unpleasant, including thick nasal mucus, stuffy nose and congestion, facial pain, headache, cough, fever, and abnormal (dysnosmia) or loss of (hyposmia/anosmia) smell [10]. The hyposmia/anosmia that occurs in sinonasal diseases is sometimes attributed to the inability of odorant molecules in the air to reach the olfactory sensory neurons (OSNs) in the OM due to increased obstruction in the nasal cavity. However, further evidence suggests that the loss of olfaction in these conditions may also be a result of direct damage to the OM, particularly the OSNs [11]. In severe cases, sinonasal diseases may lead to intracranial infections, asthmas, or smell disorders, and these factors may contribute to an increased rate of morbidity and mortality [12]. As these conditions are quite common and can impair an individual’s health and daily functioning, sinonasal diseases have the potential to significantly decrease an individual’s quality of life.

It is well understood that inhalation of harmful volatile agents can often have severe pathological consequences on the OM as it is a tissue with direct exposure to the environment. There are many studies which investigate the effect of inflammation within the nasal cavity using animal models (typically rodents) of human sinonasal diseases. In these studies, inflammation is induced either by intranasal (i.n.) administration of fungal [13–19], viral [20], or bacterial [21–25] derivatives, or through the use of transgenic models [26]. Although we do not extrapolate upon the research from these studies in this review, the overall consequences of environmental toxicant-induced immune responses in the OM is summarized in the following review [27]. Until more recently, however, the consequences of inflammation in the nasal cavity on olfactory structures of the central nervous system (CNS) including the olfactory bulb (OB) and olfactory cortex (OC) were not well understood. Here, we summarize the findings of studies investigating the pathological consequences of nasal inflammation on the OB. The authors apologize to those whose work was not included here due to space limitations.

## Organization of the olfactory bulb

The OB is a bulb-shaped nervous tissue, in humans being located on the ventral anterior aspect of the forebrain in a region known as the olfactory surface, while it is located anterior to the forebrain in mice. This small brain structure is responsible for relaying all olfactory information received by OSNs to the OC. Unlike other sensory systems, olfactory information bypasses the thalamus before reaching its primary sensory cortex; the OB functions as the relay center for olfactory information and directly transmits the information to the various components of the OC including the anterior olfactory nucleus, olfactory tubercle, piriform cortex, and entorhinal cortex [28, 29]. Although in humans the OB makes up only ~ 0.01% of the brain by volume [30], rodents rely more heavily on olfaction as the rodent OB makes up ~ 2% of the brain by volume [31].

**Fig. 1:**
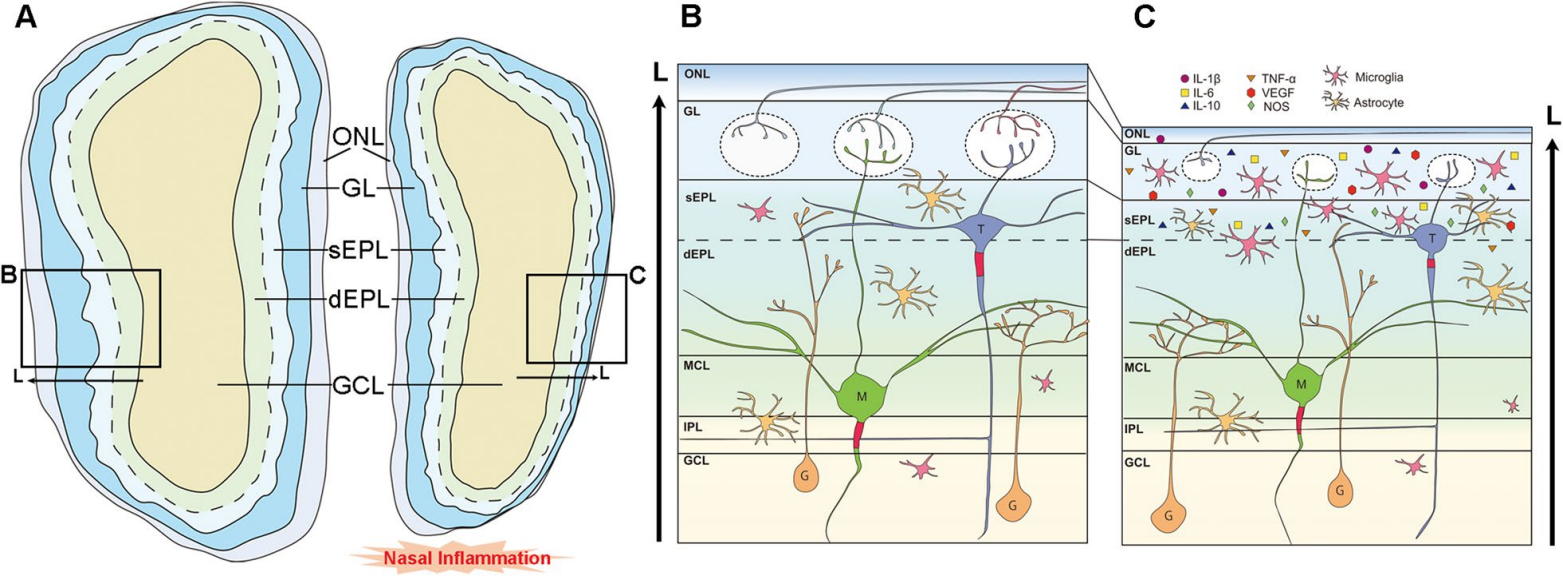
Schematic diagram of the olfactory bulb in response to chronic nasal inflammation. **A** Chronic nasal inflammation induces atrophy of the olfactory bulb (OB). The superficial OB layers including the olfactory nerve layer (ONL), glomerular layer (GL), and superficial external plexiform layer (sEPL) become thinner in response to nasal inflammation. The deeper layers of the OB including the deep EPL (dEPL), mitral cell layer (MCL), internal plexiform layer (IPL), and granule cell layer (GCL) do not change in thickness in response to nasal inflammation. Orientation of the lateral (L) region of each olfactory bulb is indicated by the arrows. **B, C** OB neurocircuitry following challenge of chronic nasal inflammation. Multiple anatomical, cellular, and molecular changes take place in the OB following nasal inflammation. Fewer OSN axons are present in the ONL and GL due to their degeneration. This loss of OSN input to the OB is a significant factor in the shrinkage of the glomeruli. The ONL, GL, and sEPL all become significantly thinner. Glial cells such as microglia and astrocytes are more prevalent and begin to activate in these layers in response to chronic inflammation. Various pro- and anti-inflammatory cytokines begin to accumulate in the superficial layers. Fewer dendrodendritic synapses are present in the sEPL. Tufted (T) cell lateral dendrites retract throughout the sEPL and the axon initial segments of T cells become fewer in number and shorter in length, while mitral (M) cells are largely unaffected. Orientation of the lateral (L) region of each olfactory bulb is indicated by the arrows on to the left and right of figure **(B)** and **(C)**, respectively. G: granule cell

The OB is divided into multiple layers in an onion-like structure, each layer consisting of unique cell types (Fig. 1). The outermost layer of the OB is known as the olfactory nerve layer (ONL). In this layer, axons of OSNs tangentially cover the surface of the OB and the OSN axon terminals penetrate to the layer just beneath, the glomerular layer (GL). The GL comprises thousands of spherical structures known as glomeruli (glomerulus, singular). Each glomerulus contains the synaptic connections between the OSN terminals, various juxtaglomerular neurons, and the primary dendrites of the OB projection neurons, mitral and tufted cells (MCs and TCs, respectively). The OB projection neurons each reside in distinct layers. For example, TCs are dispersed throughout the external plexiform layer (EPL), the layer just beneath the GL. MCs, however, exist in a single cell layer known as the mitral cell layer (MCL). Interestingly, various interneuron cell types also exist throughout the OB layers including those immunoreactive for calbindin (CB), calretinin (CR), somatostatin (SST), parvalbumin (PV), vasoactive intestinal peptide (VIP), and corticotropin releasing factor (CRF). One specific category of interneurons, however, are the granule cells which reside in the deepest layer of the OB appropriately named the granule cell layer (GCL).

Although MCs and TCs are both glutamatergic (excitatory) projection neurons that share many common cellular features in olfactory processing, MCs and TCs have important distinctions separating them as different cell types. Anatomically, TC somata are located in the EPL and extend their lateral dendrites throughout the sEPL, whereas MC somata exist in the MCL and extend their lateral dendrites in the dEPL [32–34]. Moreover, MCs and TCs differ in their axonal projection patterns; TCs target the anterior portion of the OC including the anterior olfactory nucleus, olfactory tubercle, and anterior piriform cortex, whereas MCs target the entire OC including the posterior piriform cortex, entorhinal cortex, and periamygdaloid cortex [33–36]. MCs and TCs also differ in their physiological response to glomerular stimulation; for example, the firing frequencies of MCs are significantly lower than that of TCs [37, 38]. The anatomical and physiological differences between MCs and TCs suggest that their response to a state of chronic inflammation may also differ based on divergent cellular properties.

## Effects of inflammation of the nasal cavity on the olfactory bulb

### Alterations to olfactory bulb anatomy in animal models of nasal inflammation

There are many studies aimed at investigating the effect that inflammation of the OM and OSN ablation has on the CNS. Various environmental agents and toxicants can be used to model the inflammation present in sinonasal diseases. For example, some studies utilize a method of i.n. irrigation of zinc sulfate (ZnSO_4_) in various animal models to ablate odorant receptors and OSNs in the OE [39–41]. It has been shown that rodents experience severe olfactory deficits following i.n. ZnSO_4_ irrigation [42, 43]. In zebrafish, the use of i.n. ZnSO_4_ irrigation induces inflammation of the OE [40]. Examining the impact of ZnSO_4_ beyond the nasal cavity, Burd et al. observed damage taking place to the olfactory nerve (axons of the OSNs projecting through the cribriform plate to the OB), and within the OB itself [39]. According to the investigators, axons of the olfactory nerve as well as the axon terminals targeting the OB degenerate as early as one day following ZnSO_4_ irrigation. Furthermore, it was found that the two most superficial OB layers, the ONL and GL, were significantly thinner than saline-treated controls. This suggests that damage to the OSNs is not isolated to the OE in the peripheral olfactory system, but can also be observed at the level of the OB in the CNS.

Rather than utilizing chemicals to induce inflammation of the OE, there are studies that utilize derivatives of microbial pathogens such as Satratoxin G [15, 17], roridin A [13, 16], polyinosinic:polycytidylic acid [Poly(I:C)] [20, 44], and lipopolysaccharide (LPS) [21, 25, 45–48] to better elucidate the etiological factors of immune responses in sinonasal diseases. Some sinonasal diseases including rhinitis, sinusitis, and rhinosinusitis can be primarily attributed to viral and/or bacterial infections [49]. Therefore, the administration of the aforementioned microbial derivatives in animals is a useful technique in modeling these conditions and investigating anatomical changes to the OB following the induced nasal inflammation. One particular research study administered Satratoxin G, which is a mycotoxin typically found in the black mold derived from *Stachybotrys chartarum*, to the mouse nose [15]. After just 7 days post-instillation (dpi), significant atrophy of the ONL and GL can be observed. The impact of i.n. administration of LPS on the mouse olfactory system has been more extensively investigated [21, 45–48]. LPS is an endotoxin found on the cell surface of Gram-negative bacteria and interacts with the pattern recognition receptor, toll-like receptor 4 (TLR4), of various host cells. In most of these studies, LPS was administered multiple times per week over the course of up to 3, 10, 18, or 24 weeks. The longer duration of administrations is a useful technique to replicate chronic inflammation of the OM (or chronic olfactory inflammation, COI) which can present in chronic cases of sinonasal diseases [50]. In one of these studies, the intensity of olfactory cell adhesion molecule (OCAM) was analyzed in the OB as this is a protein selectively expressed by a subpopulation of OSNs projecting from the ventral zone of the OE to the OB [51]. The expression of OCAM is significantly reduced in the GL of the OB after 3 weeks of i.n. LPS administration, suggesting that the axonal projections of OSNs to the OB are weakened or degenerated in response to COI [21]. These findings are similar to those of the studies conducted using ZnSO_4_ and Satratoxin G in which thinning of the ONL and GL is also observed [15, 39]. Therefore, it is plausible to assert that the thinning of these layers can be partially attributed to the loss of OSN axonal projections and innervation in the OB. Also, the volumetric size of the OB is significantly reduced in the LPS-treated animals when compared to saline-treated controls. This OB atrophy is first observed after 10 weeks of LPS administration, but persists and becomes more severe after 24 weeks of LPS administration [45]. In addition, unlike the ZnSO_4_ experiments, the EPL is significantly thinner in the LPS-treated OB compared to saline-treated controls [45]. More specifically, the superficial EPL (sEPL) is the primary region of EPL shrinkage, whereas the deep EPL (dEPL) appears to be unaffected as it does not change in thickness [46]. Collectively, this information suggests that COI can result in atrophy of the OB via thinning of the ONL, GL, and sEPL.

One unique characteristic about the rodent olfactory system is that there are several molecules differentially expressed by the OSNs in the dorsal and ventral zones of the OE, including OCAM, olfactory specific medium-chain acyl-CoA synthase (O-MACS), and NAD(P)H quinone dehydrogenase 1 (NQO1) [51–53]. Following 10 weeks of i.n. LPS administrations in mice, there is an equal level of OSN degeneration in both the NQO1-positive and NQO1-negative zones of the OE. However, following a 10-week recovery period of no treatment, a complete regeneration of OSNs can be observed only in the NQO1-positive region of the OE, whereas OSNs of the NQO1-negative OE undergo incomplete, or partial, regeneration [45]. Beyond the OE, the OB atrophy that takes place following the 10-week LPS paradigm first occurs via thinning of the EPL in the regions that are innervated by NQO1-negative OSNs and the thinning is delayed in the NQO1-positive innervated OB regions. Although the function of the regional differences in OSN molecular expression of the OE zones is not well understood, it is hypothesized that the cellular and molecular differences between the dorsal and ventral zones of the OE may be responsible for varying responses to LPS exposure. NQO1 is a highly inducible molecule which significantly contributes to a cell’s ability to adapt to stress through its antioxidant properties [54]. It is possible that regions of the OE containing NQO1-positive OSNs are more adequately equipped to resist the cellular degeneration resulting from LPS-induced COI, and thus the effects of COI on the OB may vary based on differences in NQO1 OSN innervation. However, since LPS is unique to bacteria but not other pathogens, such as viruses, the mechanisms by which NQO1 can be utilized to potentially protect a cell or tissue from other pathogens may be different.

### Olfactory bulb immune and inflammatory response

Microglia and astrocytes are glial cells which contribute to immune responses of the CNS via functions such as phagocytosis and secretion of cytokines and chemokines [55, 56]. I.n. irrigation of ZnSO_4_ induces significant hypertrophy of astrocyte processes within the ONL and GL of the OB [39]. In this scenario, it is hypothesized that the astrocytes may be responsible for removing degenerated OSN axon terminals. ZnSO_4_ irrigation also results in the proliferation of phagocytic cells identified as activated microglia within the same two OB layers. Similarly, i.n. administration of Satratoxin G causes infiltration of various phagocytic cell types into the OB, including neutrophils and monocytes [15]. The infiltration of these cells is observed three days after a single administration of Satratoxin G. I.n. administration of Poly I:C, a viral pathogen-associated molecular pattern, also results in an activation of both microglia and astrocytes in the OB [44]. Damage of the OE by i.n. Triton-X irrigation followed by inoculation of the opportunistic pathogen, *Staphylococcus aureus* (a bacterial species typically incapable of damaging the OB) also results in microglial infiltration to the superficial OB layers suggesting that some pathogens may be able to infiltrate the CNS following damage to the OE [22]. It has been shown that a single i.n. administration of LPS results in the accumulation of inflammatory monocytes, microglia, astrocytes, neutrophils, and even T cells along the ONL at 12 and 24 h after the administration [21, 48]. Microglia and astrocytes are also observed in the superficial OB layers (ONL, GL, and EPL) following i.n. LPS administration after one dpi [21]. The activation and hypertrophy of these glial cells persists in more chronic conditions of nasal inflammation between 3 and 24 weeks in which the cells appear to be most active after 24 weeks of LPS administration [45]. Activation of immune cells in the superficial layers of the OB may be important in preventing nasal inflammation from propagating to the CNS.

Olfactory ensheathing cells (OECs) are an additional glial cell type unique to the olfactory system which exist in the OE and lamina propria but also in the ONL of the OB [57, 58]. OECs express TLR2/4 and are therefore capable of responding to bacterial ligands such as LPS [24, 59]. When activated by olfactory nerve damage, OECs become a major phagocytic cell and promote an inflammatory response by releasing cytokines and chemokines such as IL-6, NF-κβ, TNF-α, and iNOS, and recruiting immune cells to the site of injury [22, 59–65]. Interestingly, OECs in the ONL were found to express CCL2, the primary ligand which initiates the activation of C–C chemokine receptor 2 (CCR2) in immune responses, just 12 h following a single i.n. administration of LPS [48, 66]. CCR2-positive immune cells are also distributed throughout the ONL and are closely localized to the CCL2-positive OECs, suggesting that OECs in the OB may be the primary cell type responsible for recruiting immune cells during a state of inflammation. However, since OECs wrap around the axon bundles of OSNs as they project from the OE through the cribriform plate and terminate in the OB, this data could also suggest that OECs activated by inflammation in the nasal cavity may be responsible for propagating the inflammatory response along the olfactory nerve to the OB. Although fewer studies have been conducted to investigate the response of OECs to chronic nasal inflammation, this area of research is very interesting and worth pursuing to unveil information that could provide potential therapeutic benefits for COI.

The activation of immune cells in various bodily tissues typically results in the production of cytokines, regulatory proteins which act as intercellular mediators utilized to modulate an immune response. Some cytokines are pro-inflammatory, meaning they function to activate and/or recruit immune cells, increase expression of necessary proteins such as adhesion molecules, or even induce cell death (apoptosis) [67]. In contrast, other cytokines are anti-inflammatory which can block NF-κB signaling or block the activation of lymphocytic and monocytic phagocytosis to ultimately suppress the immune response [68]. In response to a single i.n. treatment of Satratoxin G, elevated mRNA expression of the pro-inflammatory cytokines TNF-α, and IL-6 and of the chemokine MIP-2 are observed in the OB [15]. Similarly, a single administration of Poly I:C upregulates mRNA and protein expressions of pro-inflammatory cytokines including IL-1β, IL-6, TNF-α, and IFN-γ, in both the nasal cavity and the OB [44]. A single i.n. administration of LPS also upregulates expression of pro-inflammatory cytokines TNF-α, IL-1β, and IL-6, as well as anti-inflammatory cytokines IL-10 and TGFβ in the OB [48]. Furthermore, chemokines responsible for recruiting monocytes and neutrophils such as CCL2 and CXCL1, respectively, are observed in the superficial OB layers after this single LPS administration [48].

A transcriptome analysis demonstrated that early stages of COI (4 weeks of i.n. LPS administration) results in a significant upregulation of pro-inflammatory mediators collectively involved in neuroinflammation signaling pathways in the OB following LPS treatment [47]. It is hypothesized that inflammation of the OB at this stage may be orchestrated by IFN-γ-mediated signaling cascades. Interestingly, after 10 weeks of LPS administration, mRNA concentrations of pro-inflammatory cytokines including TNF-α, IL-1β, and IL-10 are significantly upregulated in the OB [46]. Collectively, these findings suggest that a significant immune or inflammatory response takes place in the OB following damage to the OSNs in the OE.

Air pollution has also been shown to induce an inflammatory state in the OB upon inhalation [69]. Mice exposed to combustion smoke for a 60 min period were analyzed 3 and 24 h post-exposure and presented with heightened levels of pro-inflammatory cytokines such as TNF-α, IL-1β, and IL-12 in the OB [69]. Factors beyond inflammation were also addressed in this study; most notably, the loss of blood–brain barrier (BBB) integrity in the OB was observed via rhodamine-B-isothiocyanate (RITC) extravasation analysis. Various molecules that are responsible for maintaining appropriate cell permeability such as VEGF, iNOS, eNOS, and nNOS were significantly upregulated in the OB. It is also worth mentioning that microglia can phagocytose astrocytic end-feet during states of inflammation in neural tissues, ultimately increasing BBB permeability [70]. These BBB impairments can allow for extravasation of immune cells and mediators to reach the affected tissue, suggesting that damage to the BBB is likely linked to a heightened state of inflammation in the OB.

### Consequences of OB inflammation on different olfactory bulb cell types

As mentioned previously, the OB cytoarchitecture is organized based on the topographical map created by the layering structure; i.e., specific cell types are found in specific OB layers. For example, periglomerular cells are a broad category of local OB interneurons located in the GL which form synapses with OSN axon terminals in each glomerulus. Periglomerular cells can be subcategorized into multiple cell types based on unique molecular expression patterns [29]. One example of periglomerular cells, which are essential for processing olfactory information, is the tyrosine hydroxylase (TH)-positive subtype [71]. In the LPS-treated mouse, the periglomerular cell TH signal is significantly reduced after only 3 weeks of i.n. LPS administration [21]. This phenotype is observed primarily in the lateral portion of the LPS-treated OB, and becomes more severe in mice treated chronically with LPS for a duration of 10 weeks [46].

A terminal deoxynucleotidyl transferase dUTP nick end-labeling (TUNEL) assay can be used to assess the impact of nasal inflammation on cell death in the OB. As such, TUNEL-positive cells are observed in the ONL, GL, and GCL following i.n. LPS administration for 10 weeks suggesting that apoptosis takes place in these layers [45]. However, the number of apoptotic cells is not significantly different between the LPS-treated OB and controls, and no TUNEL-positive cells are observed in the EPL or MCL [45]. Furthermore, interneurons including the PV- and SST-positive cells which exist preferentially in the sEPL and dEPL, respectively, do not decrease in number following 10 weeks of i.n. LPS administration [47]. Collectively, these data suggest that COI does not induce an upregulation of apoptosis for the OB projection neurons or interneurons.

The primary dendrites of MCs and TCs form dendrodendritic synapses with various interneurons within the OB including interneurons of the EPL, periglomerular cells, and granule cells [29, 72, 73]. At the presynaptic site of the projection neuron dendrites, vesicular glutamate transporter 1 (vGluT1) is highly expressed at these dendrodendritic synapses [72]. Interestingly, following 3 weeks of i.n. LPS administrations, the GL and EPL of the OB ipsilateral to the LPS-treated naris expresses significantly less vGluT1 compared to the contralateral OB or to saline-treated controls [21]. Similarly, expression of 5T4, a transmembrane glycoprotein uniquely expressed by GCs with dendrites projecting to the sEPL [74, 75], is also significantly decreased throughout the sEPL of the OB ipsilateral to the LPS-treated naris after 3 weeks of administrations [21]. These data suggest that intrabulbar neural circuits may undergo synaptic pruning in response to COI in the form of decreased dendrodendritic synapses.

The differences in the response of i.n. LPS administration between MCs and TCs was recently investigated [47]. It is important to note that, although MCs and TCs are both glutamatergic projection in the OB which receive input from OSNs, these cells are anatomically and physiologically distinct. The location of the MC somata is the MCL whereas TC somata exist in the EPL [29, 73]. The location of the lateral dendrites of these neurons is also different. Lateral dendrites extend tangentially within the EPL and form dendrodendritic synapses with surrounding cells to modulate olfactory information prior to it reaching the OC for perception. The lateral dendrites of TCs preferentially exist in the sEPL and those of MCs in the dEPL. This is an important distinction based on the topographical organization of OB layer shrinkage being isolated to the superficial OB layers in response to nasal inflammation, as was discussed previously. A significant shortening of TC lateral dendrites is observed in the OB ipsilateral to the LPS-treated naris as compared to the contralateral (control) OB after 10 weeks of i.n. LPS administration [47]. This result suggests that TCs, rather than MCs, undergo morphological impairments to their dendrites which may also indicate improper cellular communication and information processing. Furthermore, overall neuronal activity is reduced in TCs but not in MCs following the same LPS paradigm. The axon initial segment (AIS), which is the portion on the neuron’s axon most proximal to the axon hillock and cell soma and functions to maintain a neuron's polarity and initiates action potentials [76], is significantly shorter in the lateral OB for TCs but not for MCs [47]. There are also significantly fewer TC AISs present in this OB region [47]. It has been determined that shortening of the length of a neuron’s AIS is correlated to a decrease in the neuron’s function including its excitability [76–78]. This evidence collectively supports the hypothesis that TCs undergo functional impairments in response to COI.

### Recovery of the olfactory bulb

It can be challenging for an individual to experience olfactory deficits for long periods of time as a result of chronic sinonasal diseases such as chronic rhinosinusitis (CRS). Symptoms can be prolonged depending on severity of the disease. Patients can benefit from olfactory training and testing following diagnosis of CRS to help improve their olfactory functioning [79]. In some cases, however, surgery is the only option to restore olfaction [80]. The clinical findings of CRS suggest that persistent inflammation of the nasal cavity will accompany persistent olfactory deficits. However, regeneration of OSNs does occur in mice after exposure to the inflammatory mediator (e.g., LPS) is prevented [21]. Although incomplete, restoration of OSNs in the OE following nasal inflammation suggests that a recovery to the OB impairments may potentially take place. In fact, when mice are intranasally administered LPS for 10 weeks and then housed without treatment for an additional 10-week period, a recovery phenomenon takes place in the OB [45, 47]. A 10-week no-treatment period following COI reverses the atrophy of the OB to which the tissue is restored to its original size prior to treatment [45]. In particular, the thickness of the ONL, GL, and sEPL is restored. At this time point, the overall length of TC lateral dendrites is equal to (and potentially greater than) the length of lateral dendrites in the control OB [47], suggesting that TC-integrated intrabulbar circuits are restored. Similarly, the length and number of TC AISs are not different from controls following this paradigm. Also, the overall activity of TCs is remarkably improved following the recovery period. Collectively, this data reemphasizes the understanding that the OB possesses a unique ability to undergo neuroplastic changes in response to stress, and that OB recovery from COI is possible.

## Consequences of SARS-CoV-2 infection on the olfactory bulb

The COVID-19 pandemic has struck the world abruptly and raises many questions and uncertainties about viral infections of the olfactory and respiratory systems. It is well understood that the COVID-19 virus, SARS-C0V-2, infects the body through the airways and can induce fever, cough, shortness of breath, tiredness and fatigue, muscle aches, runny nose, and a multitude of other symptoms [81]. However, two of the most common features of COVID-19 in patients are anosmia and ageusia [82–88]. In fact, following SARS-CoV-2 infection, recovery of olfaction can be significantly prolonged and may take upwards of 2 months to return [89]. However, many patients report that the smell loss manifests as either a complete loss of smell or abnormal and distorted perception of odors (i.e. parosmia) and can last longer than 2 months [90]. This information suggests a strong clinical correlation between SARS-CoV-2 infection and olfactory dysfunction. It has also been demonstrated that the condition of CRS is associated with an increased risk for SARS-CoV-2 infection with a more severe prognosis [91].

Studies show that when rodents (hamsters) are intranasally administered SARS-CoV-2, multiple cell types of the OE become infected with the virus [92, 93]. Although there is evidence to suggest that OSNs become infected with SARS-CoV-2 following infection [94], there is also sufficient in vivo and clinical data demonstrating that SARS-CoV-2 does not directly infect OSNs [95, 96]. Rather, the virus is found predominantly in supporting cells such as sustentacular cells, horizontal cells, and globose basal cells, as well as in immune cells [92]. The cytokine storm produced by macrophages in response to the infection of surrounding nasal epithelial cells may cause damage to OSNs which would be a significant contributor in the progression of olfactory dysfunction in combination with the loss of function in the supporting cells [83].

A significant amount of speculation and controversy exists in regard to whether or not SARS-CoV-2 infection of the OM and respiratory tract can truly propagate to the OB and, subsequently, higher brain regions. Some studies conducted prior to the COVID-19 pandemic demonstrate that the human coronavirus can enter the CNS through the OB and cause inflammation and demyelination in the OB [97]. Extensive research has been produced since the onset of the COVID-19 pandemic which propose that SARS-CoV-2 is capable of infecting the CNS by crossing the neural–mucosal interface in the OM and the virus may utilize OSN axonal projections to reach the OB [93, 98]. Some lines of evidence also suggest that SARS-CoV-2 may be capable of directly entering the CNS via the olfactory system route or through mechanisms which degrade BBB integrity [98, 99]. These findings have also been experimentally investigated in a rhesus monkey model. Following i.n. administration of SARS-CoV-2, the virus can be transported to the CNS, specifically the OB, via the olfactory route and upregulate inflammatory cytokine production [100]. It is hypothesized that, once present in the CNS, SARS-CoV-2 accumulates in the cerebrospinal fluid of the dural sinuses, further propagating the immune responses due to the neuroimmune interface function of these sinuses [101]. On the contrary, however, SARS-CoV-2 infection of the OE in the hamster model in a different study demonstrated that the virus does not propagate to any part of the CNS following infection [96]. Therefore, it is still unclear as to whether or not SARS-CoV-2 can directly infiltrate the CNS via the route of the olfactory system, and there are likely many additional variables in the infection process that are not yet well understood.

It has been demonstrated that molecules involved in inflammatory pathways are upregulated in the OB following SARS-CoV-2 viral infection [96, 102]. These molecules include IL-6, CXCL10, IFN-β, and IL-1β. Microglial activation is observed in the ONL, GL, and EPL following SARS-CoV-2 infection in hamsters and this condition persists for as long as two weeks following a single infection [102]. On the other hand, astrocytic hypertrophy is significantly heightened but only in the EPL of the SARS-CoV-2 infected hamster OB [96]. Chemosensory behavioral tests such as the buried food finding test and the sucrose preference test confirm that SARS-CoV-2 infection induces acute anosmia and ageusia in the hamster model [86]. These findings strongly parallel the in vivo data for the aforementioned models of COI.

Interestingly, in the case of SARS-CoV-2 infection of the rodent, the consequences of the infection on the OB are opposite to that of LPS with respect to regional differences defined by NQO1 expression [96]. The damage to the OE is more severe in the NQO1-positive regions than in NQO1-negative regions, whereas this phenomenon is reversed following LPS-induced COI as mentioned previously [45]. Similarly, although the size of the OB is decreased in both the NQO1-positive and -negative regions following SARS-CoV-2 infection, the density of OSN axon terminal within the GL is significantly reduced in NQO1-positive but not in the NQO1-negative regions [96]. This data suggests that the response of both the OE and OB to viral and bacterial infections may utilize contrasting defense mechanisms which manifest as opposing phenotypes in the olfactory tissues.

Some hypotheses for neurodegenerative diseases such as the olfactory vector hypothesis [103–105] suggest that agents can enter the brain via the olfactory route and cause neuropathology. Although the research is still controversial, similar hypotheses have been proposed for the pathogenicity of SARS-CoV-2 infection on the CNS suggesting that infection through the nasal cavity and OE may result in infection in the CNS which may be linked to neurodegenerative processes [85]. Beyond olfactory and gustatory functions, hyper-phosphorylated-tau and alpha-synuclein proteins are observed to accumulate in the brain, particularly among suprahippocampal cortical neurons occurring two weeks after a single infection with SARS-CoV-2 [102]. This is some of the first data which connects SARS-CoV-2 infection to the pathogenesis of neurodegenerative diseases including Alzheimer’s and Parkinson’s disease. While it is possible that SARS-CoV-2 infection may be linked to neurological disorders including neurodegenerative diseases [106], this is an area of research that requires more time and studies in order to appropriately address this question.

## Conclusion

This review has summarized the impact that nasal inflammation induced by fungal, viral, or bacterial derivatives has on the morphological and physiological features of the rodent OB. Although the mechanisms by which these various microbial derivatives induce a state of inflammation in the OM involve different receptors and signaling pathways, they have similar effects on the OB, including atrophy via thinning of the superficial OB layers, activation of glial cells, and upregulation of inflammatory cytokines (Fig. 1). Furthermore, the multiple studies investigating the impact of LPS-induced COI on the OB have demonstrated functional impairments to a specific group of OB projection neurons, TCs. This data suggests that olfactory information typically received by TCs may be inappropriately processed in a state of COI and, therefore, signaling to the OC regions innervated by TCs may be diminished. Although the OB is the first relay station for olfactory information in the CNS and significant information processing takes place at the OB, the olfactory information is not completely processed until it reaches the OC [107]. As with the other sensory systems associated with the mammalian CNS, the olfactory system comprises first-, second-, and third-order neurons. Although sensory information is relayed in a streamline fashion through these neurons, a great deal of modulation takes place at each level of the network. Potential damage to the sensory system at any of these levels may significantly impair the information, ultimately halting or altering the sensation prior to CNS processing. For instance, clinical studies of spinal cord injury patients have demonstrated that the somatosensory cortex undergoes significant atrophy and alterations in brain activity in response to spinal cord injury [108–110]. Similarly, the disease of diabetic retinopathy has been shows to result in impairments to the primary visual cortex as well as cortical remapping following onset of the disease [111–113]. With respect to the olfactory system, however, few studies have been conducted investigating the role of damage to the OM on the OC. The concept that COI can induce neurological deficits in the olfactory cortex or potentially other regions of the CNS is a new and interesting area of research that has not yet been fully examined. Further research addressing the impact of COI on the higher brain regions of the CNS may provide novel insights into a route of transmission for inflammation from the periphery to the CNS.

## Data Availability

Not applicable.

## Abbreviations

AIS: Axon initial segment
BBB: Blood–brain barrier
CNS: Central nervous system
COI: Chronic olfactory inflammation
CRS: Chronic rhinosinusitis
dEPL: Deep external plexiform layer
dpi: Days post-instillation
EPL: External plexiform layer
GCL: Granule cell layer
GL: Glomerular layer
i.n.: Intranasal
IPL: Internal plexiform layer
LPS: Lipopolysaccharide
MC: Mitral cell
MCL: Mitral cell layer
NQO1: NAD(P)H quinone dehydrogenase 1
OB: Olfactory bulb
OC: Olfactory cortex
OCAM: Olfactory cell adhesion molecule
OE: Olfactory epithelium
OM: Olfactory mucosa
ONL: Olfactory nerve layer
OSN: Olfactory sensory neuron
Poly(I:C): Polyinosinic:polycytidylic acid
PV: Parvalbumin
sEPL: Superficial external plexiform layer
SST: Somatostatin
TC: Tufted cell
TH: Tyrosine hydroxylase
TUNEL: Terminal deoxynucleotidyl transferase dUTP nick end-labeling
vGluT1: Vesicular glutamate transporter 1
